# JAOS under a continuous publication workflow in 2018

**DOI:** 10.1590/1678-77572017ed003

**Published:** 2017

**Authors:** Editors and technical staff

The Journal of Applied Oral Science is pleased to announce that it will operate only under a continuous publication online modality in 2018. This model will result in annual volumes, without issue numbers, and the papers will be published as accepted and designed for publication. However, the number of manuscripts published annually will not be modified.

The volumes will be divided into the following sessions: editorials (when necessary), original manuscripts and review manuscripts, the latter being published only upon editor's invitation. Case reports (including case series and clinical protocols) will no longer be accepted.

The continuous publication system delivers content to our readers much more quickly than the old issue-based model, i.e., the paper publication will occur in individualized way. This means that manuscripts will no longer have to wait for the next issue - once they are ready to publish, they immediately go online.

The editorial board and technical team of the JAOS analyzed the criteria adopted by other journals as well as the recommendation of SciELO, which pointed out the following operational advantages to assume this publication system:

– A faster and more definitive way of communicating research results will be established, favoring authors and researchers;
– Indexing and visibility of papers in databases will occur more consistently and in less time;
– Journals can considerably shorten the time between acceptance and publication of papers, since they do not accumulate more manuscripts to publish in the traditional issue-by-issue model (PACKER et al., 2016).

In addition to the continuous publication of manuscripts, JAOS is also reviewing some points in the publication standards to meet the criteria already established by SciELO, and also to reduce bureaucracy and speed up the publishing process. The new guidelines will sooner be announced on the journal's website.
